# Endotoxin-induced m6A RNA methylation landscape in lung endothelial cells: role of METTL3 in regulating inflammation and injury during acute lung injury

**DOI:** 10.1016/j.bbadis.2025.167907

**Published:** 2025-05-14

**Authors:** Anlin Feng, Ying Liang, Panfeng Fu, Yishu Dong, Stephen M. Black, Ting Wang

**Affiliations:** a Center for Translational Science, Florida International University, Port Saint Lucie, FL 34987, USA; b Department of Environmental Health Sciences, Florida International University, Miami, FL 33199, USA; c Department of Cellular and Molecular Medicine, Florida International University, Miami, FL 33199, USA

## Abstract

Acute Lung Injury (ALI) involves diffuse alveolar damage, neutrophil infiltration, and pulmonary edema, with unacceptable mortality. Bacterial lipopolysaccharide (LPS) activates inflammatory pathways in ALI, which are then regulated by transcriptional and post-transcriptional pathways to affect gene expression. RNA methylation, N6-methyladenosine, is the main m6A mRNA modification that controls the expression of various genes in different environments. There are very few facts about LPS’s effect on m6A RNA methylation. This study will explore the m6A RNA methylation landscape in lung endothelial cells (ECs) to understand its role in lung inflammation. In this study, lung endothelial cells were treated with LPS, and the dynamics of mRNA m6A methylation were examined through m6A-methylated RNA sequencing. RNA abundance was measured with RNA-seq, and global protein expression and m6A-binding proteins were identified using mass spectrometry (MS). Following LPS treatment, global m6A methylation levels increased along with the upregulation and nuclear translocation of METTL3 protein, while demethylase activity remained unchanged. METTL3 drove LPS-induced m6A methylation and endothelial injury, as shown by selective METTL3 siRNA and the inhibitor STM2457. MeRIP-seq analyses revealed increased m6A sites near the 5’ UTR in LPS-treated cells, with m6A methylation correlating positively with gene expression. The metabolic and apoptosis pathways were shown to be more enriched in different types of methylated exons. METTL3-mediated m6A methylation targeted inflammatory genes, enhancing protein expression in chemokine signaling and MAPK pathways. STM2457 effectively mitigated LPS- or CLP-induced experimental ALI. According to this paper, LPS-mediated m6A RNA methylation is described in terms of genomic structure. Modulation of m6A methylation exerts influence over LPS-mediated endothelial gene expression and the ensuing inflammatory response.

## 1. Introduction

Patients who have sepsis, which is a potentially fatal organ dysfunction, are clinically symptomatic of the condition. One of the biggest challenges of therapy design is the deficient interpretation of sepsis pathobiology. Endotoxin, also called lipopolysaccharide, is the most important mediator of inflammation that is linked to the development of sepsis [1]. LPS-induced gene expression alterations drive persistent inflammation within the lungs, exacerbating tissue damage and compromising respiratory function [2]. This sustained inflammatory response, orchestrated by LPS-mediated gene expression, significantly contributes to the morbidity and mortality associated with sepsis-induced ALI. As such, understanding the mechanisms by which LPS influences gene expression and perpetuates inflammation is crucial for the development of effective therapeutic interventions targeting sepsis-induced ALI.

Recently, it has been discovered that RNA modifications play a crucial role in regulating gene expression and translation. *>*170 RNA modifications have been found for decades, but only a few RNA modifications can regulate the turnover and translation of transcripts [3]. Modification of N6-methyladenosine (a significant regulator of gene expression in mammalian cells) was found for RNA methylation [4]. The protein complex comprising methyltransferase-like 3 (METTL3) and 14 (METTL14), along with their splicing regulator Wilms’ tumor 1-associated protein (WTAP), was responsible for modifying m6A methylation. This protein complex can detect and transfer a methyl group to these m6A methylation sites [5]. m6A RNA methylations are reversible, and they can be removed by demethylases, including the obesity-associated protein (FTO) [6] and AlkB homolog 5 (ALKBH5) [7]. The intricate balance of m6A RNA methylation homeostasis is governed by the co-ordinated actions of methyltransferases (writers), which add methyl groups, and demethylases (erasers), which remove methyl groups. The specific control of m6A changes within RNA molecules is achieved through this dynamic interaction. The m6A RNA methylation can be detected and bound by various m6A binding proteins (readers). Selective binding partners will impact RNA fate, including RNA splicing [8], mRNA translation efficiency [9], as well as mRNA stability [10]. Some studies suggest that m6A RNA methylation regulation might play an important regulatory role in the pathogenesis of carcinogenesis [11] and inflammation [12]. However, until now, the physiological impact of m6A methylation homeostasis, as well as the pathobiology of altered m6A RNA methylation, is largely unknown.

In this study, the impact of the LPS challenge on mRNA m6A modification in lung cells is defined. The kinetics and characteristics of mRNA m6A modification in lung endothelial cells are explained, which are then subjected to methylated RNA immunoprecipitation sequencing (MeRIP-seq). A new LPS-driven and METTL3-dependent m6A RNA methylation landscape is fully characterized. We have uncovered that m6A modification has a strong relationship with proinflammatory gene expression, and attenuation of m6A methylation by METTL3 inhibition is a novel therapeutic strategy for experimental ALI.

## 2. Materials and methods

### 2.1. ECs culture

Human pulmonary artery ECs were cultured in EGM-2 endothelial cell growth medium (NC9525043, Lonza, Switzerland) with 10 % fetal bovine serum (A5256701, Thermo Fisher, U.S.) at 37 ^◦^C in a 5 % CO_2_ humidified atmosphere. The ECs were treated with LPS (L2630, Sigma-Aldrich, Burlington, U.S.) for 4, 8, 24, and 48 h (100 ng/mL). Particularly, this Lonza HAPEC (category number CC-2530) were used for passages 6–9 for experiments, and tested negative for mycoplasma, bacteria, yeast, and fungi.

### 2.2. m6A dot blot

Total RNA from cultured cells was obtained using an RNA extraction kit (74,704, QIAGEN, Germany) following the manufacturer’s procedure and dissolved in RNase-free water (AM9916, Thermo Fisher, U.S.). Then, samples were incubated for five minutes at 80 ^◦^C, cooled on ice right away following denaturation. For five minutes, RNA samples were immediately loaded onto the Hybond-N+ membrane (RPN82B, GE Healthcare, Chicago, U.S.) and cross-linked with a UV cross-linker. Following incubation in blocking buffer (5 % milk) for one hour, the prepared membrane was gently shaken at 4 ^◦^C in anti-m6A antibody (ab151230, Abcam, UK). The membrane was washed with TBST and incubated with IgG-HRP antibody (31,460, Thermo Fisher, U.S.) for 1 h at room temperature with gentle shaking. The protein bands were detected by ECL reagent (Cell Signaling, Danvers, U.S.).

### 2.3. Western blot analysis

The whole cell lysis buffer (1× RIPA Buffer) was used to resuspend the cells, and the mixture was supplemented with 1 mM DTT, protease, and phosphatase inhibitor cocktail (78,440, Thermo Fisher, Waltham, U. S.). The concentrations of protein were measured by dye-binding assays (5,000,201, Bio-Rad, Hercules, U.S.). The sample buffer containing SDS-PAGE was added to the protein samples, and then DTT was added, and the mixture was boiled for 10 min. On the SDS-PAGE gels, 10–20 μg of protein was separated, and the resulting protein was then blotted onto the Immun-Blot PVDF membrane (Bio-Rad, Hercules, U.S.). Blocking buffer was used to block membranes, and the membranes were then covered with the primary antibody and then the secondary antibody. The membranes were treated with ECL Western Blotting Substrate (32,106, Thermo Fisher, Waltham, U.S.), and the bands were captured using the Invitrogen iBright Imaging Systems (Thermo Fisher, Waltham, U.S.).

### 2.4. MeRIP-seq and RNAseq

Using the RNeasy Plus Mini Kit (74,134; QIAGEN, Düsseldorf, Germany), total RNA was extracted. The m6A methylated RNAs were pulled down by the EpiQuik CUT&RUN m6A RNA Enrichment (MeRIP) Kit (P-9018–24, EpiGentek, NYC, U.S.). The next-generation sequencing approaches used in this study were all carried out by the UA RNA-sequencing lab. The data of MeRIP- and RNA-seq results were cleaned and trimmed by the FastQC (Version 0.11.9) and the read trimming tool Trimmomatic (Version 0.39). HISAT2 (Version 1.0) was used to map sequencing reads to the Genome Reference Consortium Human Build 37 (hg19) [13]. The exomePeak [14] (Version.2.13) was used for peak calling. CHIPseeker [15] (Version. 3.16) was used for peak annotation, comparison, and visualization. HOMER [16] (Version 4.1) was used for motif discovery. The gene function and pathway analysis for annotated genes was implemented by DAVID [17] (Version 6.8) with an adjusted-*P* value cutoff of 0.05.

### 2.5. Proteomic analysis

The ECs were treated with LPS (100 ng/mL) for 0 and 24 h, and harvested cells from both control and LPS-treated cells into a 15 mL conical tube. Then, the cells were collected by centrifugation. *Re*-suspend an equal pellet volume of complete lysis buffer from the RNA Immunoprecipitation Kit (Sigma-Aldrich, Burlington, U.S.) and incubate the lysate on ice for 5 min. Purified anti-m6A antibody was added and combined with magnetic beads. Mix and incubate the RIP buffer with magnetic beads overnight, and wash the mixture with RIP Wash Buffer afterwards. The immunoprecipitated samples were separated on SDS-PAGE gels, and the desired bands were cut and submitted to the Taplin Biological Mass Spectrometry Facility (Boston, U.S.). The protein intensity in each group was compared, and the biological functions were summarized by GO analysis using DAVID.

The HPAECs were treated with the same treatment, and the collected cells were lysed in RIPA buffer (89,900, Thermo Fisher, Waltham, U.S.) for whole-cell protein extraction. The 6-well plate was scraped off by the HPAEC, which was then moved to a 1.5 mL microcentrifuge tube by means of a cell scraper. Keep the tubes on ice and centrifuge for 30 min. The supernatant was moved into a new 1.5 mL tube. The protein samples (at least 1–2 mg for each sample) were submitted to the Thermo Fisher Scientific Center for Multiplexed Proteomics (TCMP) for multiplex quantitative proteomic analysis. The normalized sum intensity, which is the abundance value of all the peptides in the identified protein data, was used for further analysis. The R package limma (version: 3.18) was used to detect all differentially expressed proteins.

### 2.6. Real-time PCR

The purified total RNA was obtained using the RNA isolation kit (74,134, QIAGEN, Düsseldorf, Germany). The cDNA synthesis was performed using the Reverse Transcription Kit (205,311, QIAGEN, Düsseldorf, Germany). The changes in gene expression were detected by the real-time PCR system (Thermofisher, Waltham, U.S.).

### 2.7. Murine experimental ALI model

All animal study procedures were approved by the Institutional Animal Care and Use Committee (IACUC) of the Florida International University (Protocol ID: 21–027).Two acute lung injury models were employed in this work. For the LPS-induced ALI model, male wild-type C57BL/6 mice were intraperitoneally (i.p.) treated with 15 mg/kg STM 2457 two hours before the LPS challenge (1 mg/kg, i.p.). In the cecal ligation puncture (CLP) model, male wild-type C57BL/6 mice were administered STM 2457 (15 mg/kg, i.p.) right after CLP surgery [18]. Bronchoalveolar lavage (BAL) fluid were extracted and lung tissues were harvested twenty four hours after LPS exposure or CLP surgery.

### 2.8. Bronchoalveolar lavage (BAL) fluid analysis

1 mL of PBS was injected into the BAL fluid, which was then removed using a tracheal cannula. For 20 min (4 ^◦^C), cells in the BAL fluid were pelleted at 300 x g. The supernatant was collected and centrifuged at 12,000 *g* for ten minutes. For the purpose of measuring cytokines and the assay of proteins, the supernatant was taken. Simultaneously, cell pellets from the previous centrifugation were resuspended in 200 μL of PBS and 1 mL of red blood cell lysate buffer for 10 min to lyse red blood cells. The white blood cells were resuspended in 200 μL of PBS after being pelleted at 300 ×g for 20 min (4 ^◦^C). The total number of cells was calculated using a hemocytometer.

### 2.9. Histological analysis of the mouse lung

The lung tissues were filled with 10 % formalin and subsequently processed into paraffin-embedded blocks. Sections of 4 μm were then cut and stained. The stained sections were evaluated based on several parameters: the presence of neutrophils, the presence of neutrophils in the interstitial space, and the presence of thickening in the alveolar septa, as previously reported by others. For an objective assessment of the levels of lung injury, samples were randomly renamed for a single-blinded evaluation.

## 3. Results

### 3.1. LPS alters the m6A landscape in human lung endothelial cells

**Fig. 1.**
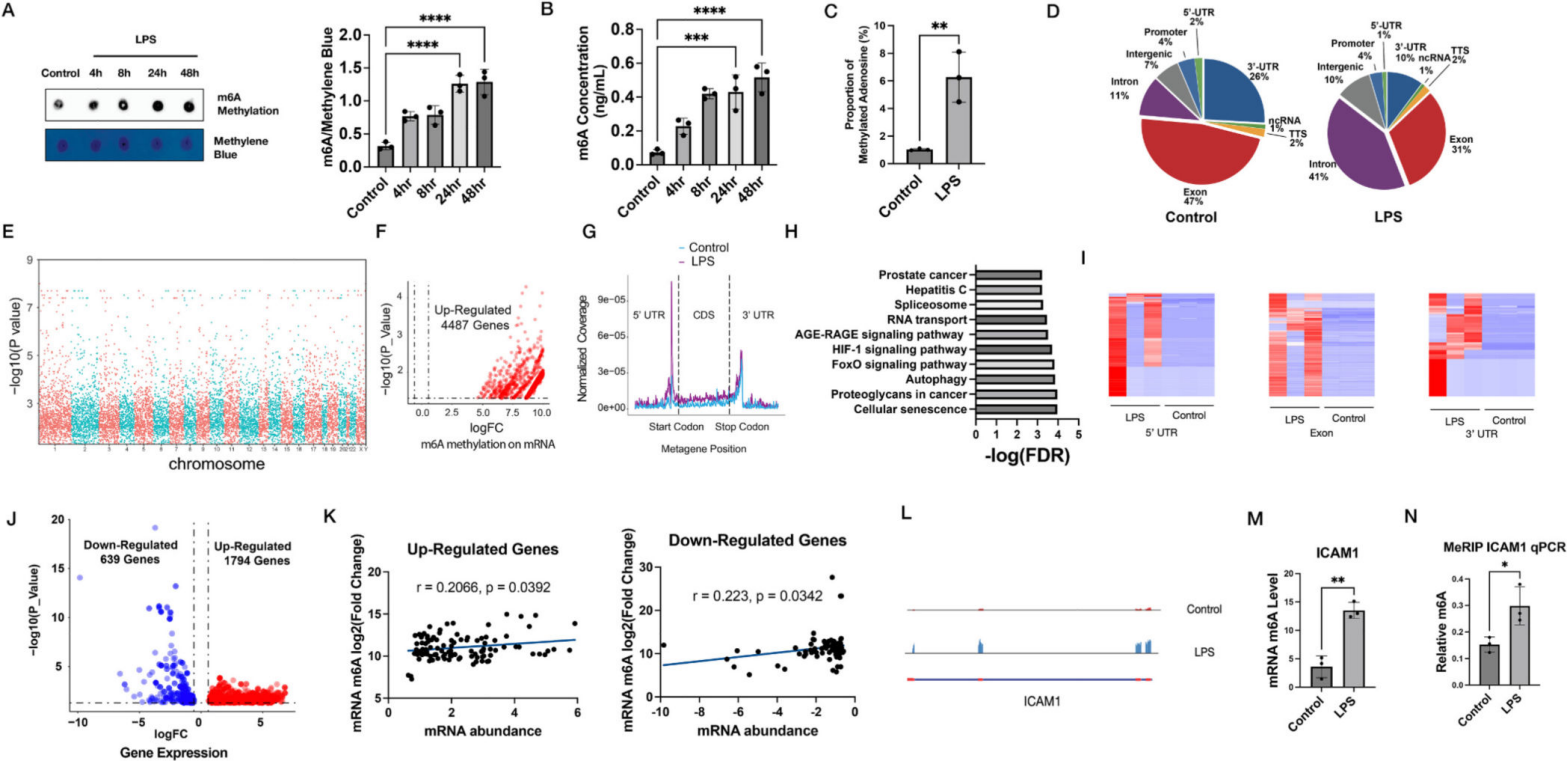
LPS increases m6A levels and results in transcript level escalation. The global RNA m6A level in ECs determined by (A) dot blot and (B) m6A ELISA; (C) m6A/A ratio was evaluated by MeRIP-Sequencing read count; (D) Genomic distribution of all m6A peaks on control (left) and LPS-treated ECs (right); (H) KEGG analysis of all up-regulated m6A peaks in LPS-treated ECs; (F) Volcano plot of all dysregulated m6A peaks in LPS-treated ECs; (G) The distribution of all control (blue) or LPS-treated (purple) m6A peaks using metagene plot; (I) Manhattan plot of genomic distribution of all m6A peaks’ corresponding *P* values; Heat-maps of m6A levels of all peaks within 5’-UTR, exon, and 3’-UTR region; (J) Volcano plot of all up-regulated (red) and down-regulated (blue) genes; (K) Correlation analysis of all up-regulated (left) and down-regulated (right) genes’ transcript levels with m6A levels; (L) Genomic visualization of the m6A peaks normalized signal for ICAM1 in ECs following treatment with LPS; (M) m6A levels of ICAM1 in control and LPS-treated ECs; (N) MeRIP-qPCR of ICAM1 in control and LPS-treated ECs. * p < 0.05, ** p < 0.01, *** p < 0.001, **** p < 0.0001.

ECs were treated with LPS (100 ng/mL) at different times and relative levels of RNA m6A methylation can be detected by m6A dot blot (Fig. 1A). The abundance of m6A methylation was increased in LPS-treated cells in a time-dependent manner starting from 4 h, and the increase peaked at 24 h (Fig. 1A). In addition, the elevated m6A RNA methylation was further confirmed by ELISA assay [19], and a peak was also found at 24 h which was consistent with dot blot (Fig. 1B).

To characterize the m6A transcriptome-wide RNA methylation pattern, we performed methylated RNA immunoprecipitation sequencing (MeRIP-seq) [20] on total RNA samples from control and LPS-treated ECs. The ratio of N^6^-methyladenosine to total adenosine (m6A/A ratio) in MeRIP confirmed the up-regulation of m6A RNA in LPS-treated ECs (Fig. 1C). m6A peaks shifted to exons after LPS treatment, from 31 % to 47 % (Fig. 1D). The heatmap of m6A peaks further verified the up-regulation of m6A in 5’UTR, 3’-UTR, and exon (Fig. 1I) further confirming the globally heightened m6A methylation upon LPS challenge.(Fig. 1E). All 4487 m6A peaks on mRNA were found with all increased abundances in LPS-treated ECs (Fig. 1F, see the list of m6A methylated RNA sites in Supplementary Table S1). These m6A methylated peaks showed a major increase in the coding sequence (CDS) region, especially in the region close to 5’-UTR in LPS-treated ECs (Fig. 1G). KEGG pathways such as RNA transport, HIF-1 signaling pathway, and autophagy were enriched among these m6A hypermethylated genes (Fig. 1H), suggesting a consistent and functional consequence of LPS challenge. The Gene Set Enrichment Analysis (GSEA) showed that the NOD-like receptor and apoptosis signaling pathways were enriched among these m6A-hypermethylated genes (Supplementary Fig. 1A–B). This confirms that LPS induces the m6A methylation of mRNAs in the pro-inflammatory pathways in endothelial cells, suggesting a vital molecular mechanism involved in the effect of LPS on regulating the expression of pro-inflammatory genes in endothelial cells. As a genomic geographic profile, the Manhattan plot revealed that m6A methylation peaks among whole-genome distributed equally among each chromosome (Fig. 1G).

To understand the transcriptional impact of LPS-induced m6A RNA methylation, we further performed transcriptomic analysis of the same cells. RNA-sequencing revealed most genes were up-regulated in LPS-treated samples (639 down-regulated and 1739 up-regulated genes, Fig. 1J, see the DEGs list in the Supplementary Table S2). To verify whether m6A methylation will affect the corresponding mRNA’ abundance, we performed a correlation analysis. It showed that both up-regulated and down-regulated m6A peaks were positively correlated with corresponding genes’ transcripts (Fig. 1K). This analysis suggests that m6A methylation level in mRNA is a marker of the transcript abundance in these LPS-challenged cells.

To validate our genome-wide findings, we picked one gene/mRNA for validation. ICAM1 is a glycoprotein typically found on the cell membrane of endothelial cells. It involves the trans-endothelial migration of leukocytes to the inflammatory sites which plays an important role in innate immunity [21,22]. ICAM1 was chosen to illustrate peaks whose m6A peaks were significantly increased in exon regions after LPS treatment (Fig. 1L), while the m6A methylation level also confirmed this result (Fig. 1M). To validate increased ICAM1 m6A methylation by LPS challenge, we pulled down all m6A-methylated RNAs (MeRIP) and confirmed the up-regulation of ICAM1 m6A level in the LPS-treated sample with qPCR (Fig. 1N).

LPS alters the m6A landscape to favor the expression of inflammatory pathway genes.

**Fig. 2.**
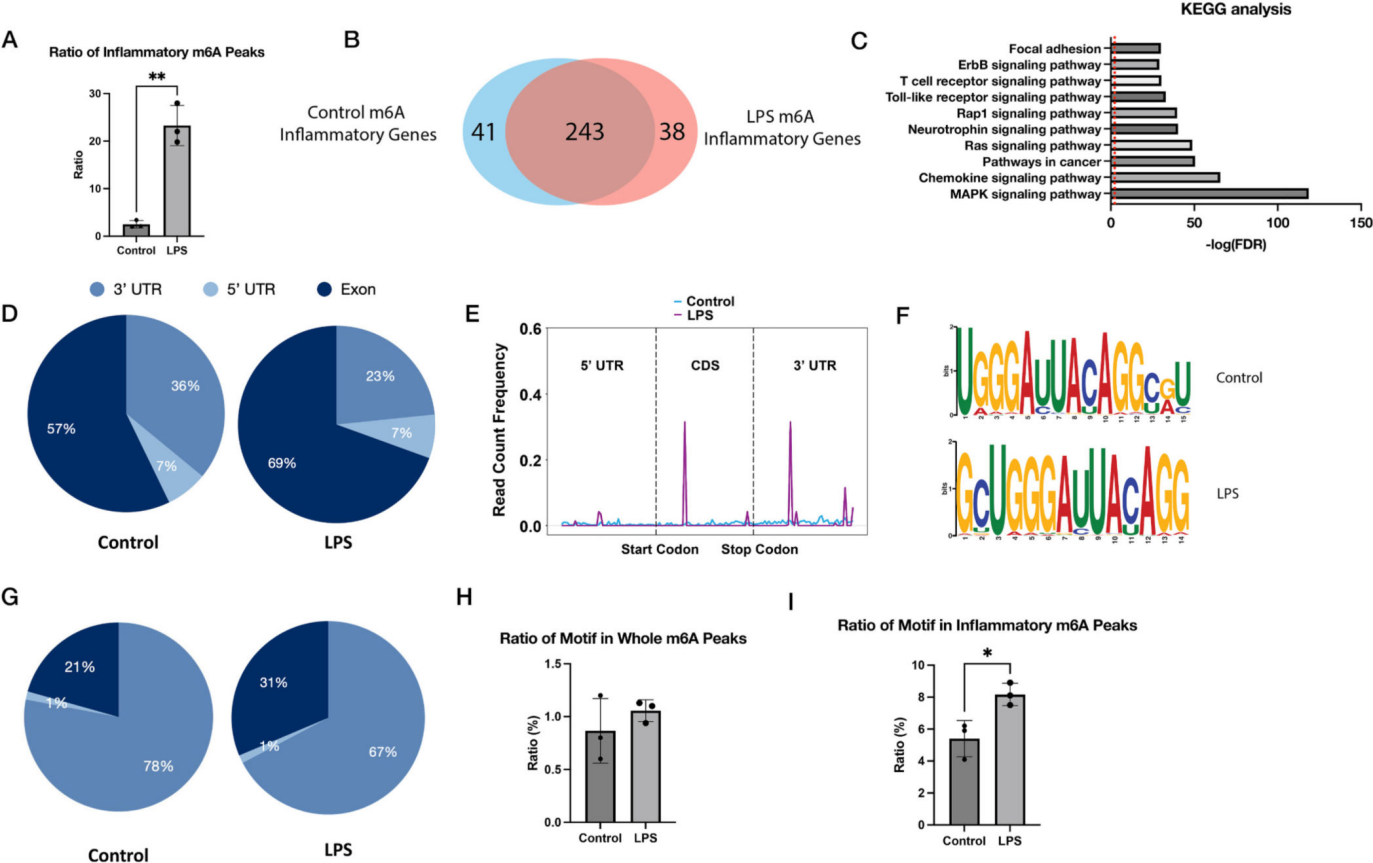
LPS alternates the m6A motif patterns in endothelial cells to favor inflammatory gene expression. (A) Proportion of m6A peaks in inflammatory-associated genes; (B) Venn diagram of the intersection of m6A methylated inflammatory-associated genes in control and LPS-treated samples and KEGG analysis of overlap gene; (C) The ratio of m6A-methylated peaks in inflammatory-associated genes among all genes; (D) Pie charts showing the distribution of m6A methylated inflammatory-associated peaks in control and LPS-treated samples; (E) The distribution of read count frequency in genomic regions around the transcription start site (TSS); (F) Motif analysis of the sequences under control (upper) and LPS-treated (down) m6A methylated peaks; (G) Pie charts showing the distribution of m6A peaks contains motifs in control and LPS-treated samples; Proportion of (H) all genes or (I) inflammatory-associated genes in motifs in control and LPS-treated m6A methylated sequences. * p < 0.05, ** p < 0.01.

The LPS incites a TLR4-dependent cellular signaling in ECs, resulting in the activation of NF-κB [23] and MAPK [24] pathways, as well as heightened production of pro-inflammatory cytokines (IL-6, IL-1β, and TNF-α). The critical role of m6A in regulating inflammatory reactions within LPS-treated ECs was examined by analyzing MeRIP-seq data, which helped establish the characteristics of pro-inflammatory responses throughout this process. The proportion of m6A peaks linked to inflammation-associated genes in cells exhibited a significant increase post-LPS treatment (Fig. 2A, see the list of inflammation-associated genes in the Supplementary Table 3). A comparison of these methylated inflammation-associated genes in both control and LPS-treated cells revealed a high degree of overlap (Fig. 2B), suggesting the control of inflammatory gene m6A methylation is thoroughly sustainable. The MAPK pathway is strongly associated with methylated genes (Fig. 2B). It was found that the upsurge in m6A peaks was primarily attributable to an increase in the number of m6A peaks per gene, a hike verified in LPS-treated cells (Fig. 2C). Additionally, LPS treatment led to a rise in m6A peaks in exons of inflammation-associated genes (Fig. 2D). The average profile of m6A peaks in the transcription start site (TSS) region is shown in Fig. 2E, with a high level of enrichment observed near the TSS for inflammation-associated m6A peaks. Under LPS treatment, inflammation-associated gene m6A sites tend to exhibit one additional peak near the TSS in the exon.

By utilizing motif analysis for m6A peaks, we found that m6A occurs mostly in the AURACA (R = U or C) consensus sequence, which is summarized from the consensus sequence in all EC samples (in both control and LPS groups, Supplemenary Fig. 1C), and it is particularly found within m6A peaks in LPS-treated and control samples (Fig. 2F). The m6A distribution analysis showed that m6A-methylated AURACA peaks are mainly enriched in 3’ UTR regions, and some regions shifted to exons after LPS treatment (Fig. 2G). LPS treatment also resulted in an increase in these motifs within inflammation-associated genes (Fig. 2H). These data suggest LPS remodeled m6A methylation profile to favor inflammatory gene expression at an m6A-RNA methylome level.

### 3.2. m6A-binding and controlling protein patterns

**Fig. 3.**
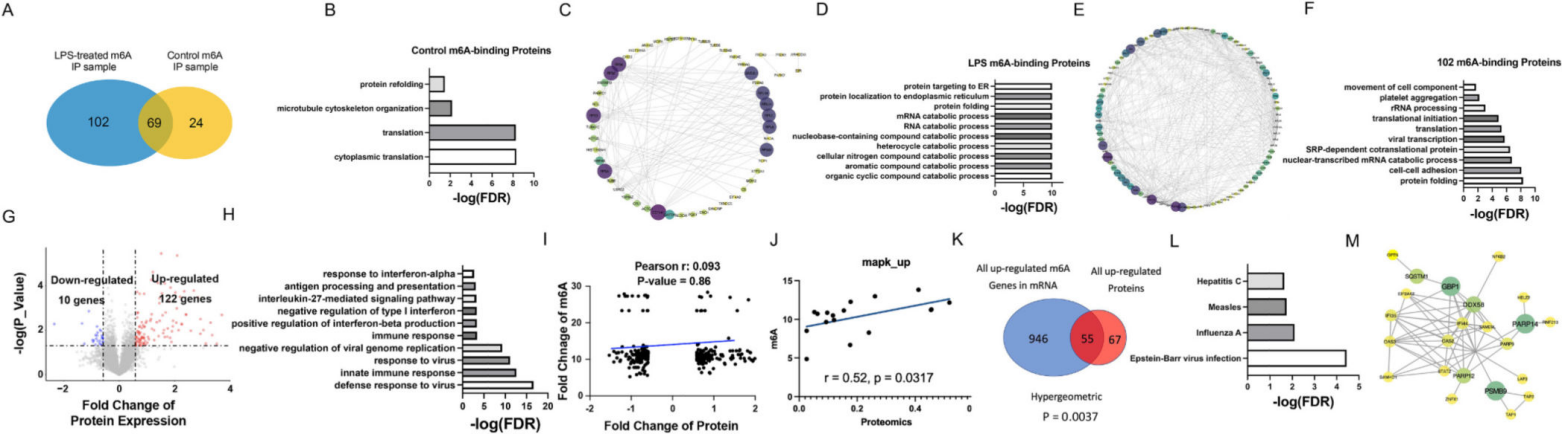
m6A-binding and controlling protein patterns. (A) Intersection of LPS-treated m6A-binding proteins with control m6A-binding proteins; B–C. GO (B) and PPI (C) network analysis of control m6A-binding proteins; D–E. GO (D) and PPI (E) network analysis of LPS-treated m6A-binding proteins; (F) GO analysis of 102 proteins in A? LPS-treated only affected?; (G) Volcano plot of all up-regulated (red) and down-regulated (blue) proteins in LPS-treated ECs; (H) GO analysis of all differentially expressed proteins; (I-J) Correlation analysis of all proteins (I) or MAPK pathway (J) protein expression with corresponding genes’ m6A levels; (K) Intersection of all up-regulated m6A genes on mRNA with all up-regulated proteins; (L) KEGG and (M) PPI network analysis of 55 proteins of K overlapped proteins.

RNA-protein complex was extracted from ECs and pulled down by m6A antibody, and all these proteins were detected by mass spectrometry [25]. For control samples, 93 m6A-binding proteins were found (Fig. 3A, see the protein list in the Supplementary Table 4), and they are highly associated with translation-related pathways (Fig. 3B–C). While in LPS-treated samples, more m6A-binding proteins were found (171 proteins, Supplementary Table 4, Fig. 3A) and they are strongly related to RNA catabolic pathways (Fig. 3D–E). Protein-protein interactions in LPS-treated samples have more intense connections than in control samples (Fig. 3C–E). 102 proteins were specifically found in LPS-treated samples (Fig. 3A). Translation and translation initiation pathways, as well as cell-cell adhesion, are enriched among these 102 proteins (Fig. 3F).

The expression levels of total protein were also determined by total proteome analysis. Most proteins in LPS-treated ECs were increased (102 up-regulated and 28 down-regulated proteins (Fig. 3G, see the protein list in the Supplementary Table 5). Viral defense pathways and immune response pathways were enriched among these 102 proteins (Fig. 3H). According to correlation analysis, global protein expressions did not correlate with corresponding genes’ m6A levels, suggesting m6A is not the only driver of protein upregulation in LPS-challenged cells (Fig. 3I). However, protein expressions in MAPK pathways were selectively positively correlated with m6A levels (Fig. 3J). The MAPK pathway is a major signaling pathway that regulates multiple functions of ECs in response to exogenous and endogenous stimuli [26–28].

55 common genes were found among all genes with up-regulated m6A and up-regulated proteins (Fig. 3K). The significant enrichment of m6A hyper-methylated genes in upregulated proteins suggested a direct contribution of m6A hyper-methylation to protein abundance in LPS-challenged ECs. Several viral pathways like Hepatitis C and Influenza A were enriched among these 55 genes (Fig. 3L). A strongly connected network was found in these 55 genes (Fig. 3M).

### 3.3. METTL3 is crucial to m6A methylation

**Fig. 4.**
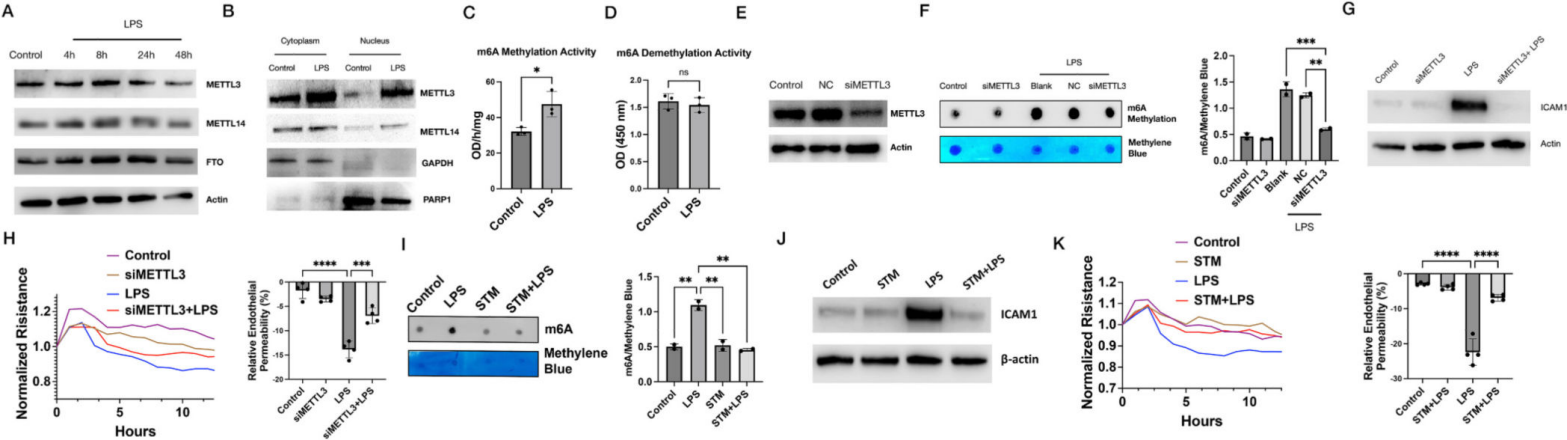
METTL3 is responsible for LPS-induced m6A methylation and functional outcome. (A) Protein expression of m6A regulators (METTL3, METTL14 and FTO) in LPS-treated cells; (B) Cytoplasm and nucleus protein expression of METTL3 and METTL14; (C) m6A methylase and (D) demethylase activity after LPS-treatment; (E) Verification of METTL3 knockdown by METTL3 siRNA; (F) Dot blot of m6A methylation in METTL3 knocked down ECs; (G) ICAM1 expression in METTL3 knocked down ECs; (H) Transendothelial resistance of METTL3 knocked down ECs; (I) Dot blot of m6A methylation in STM-treated (dose and time) ECs; (J) ICAM1 expression in STM-treated (dose and time) ECs; (K) Transendothelial resistance of STM-treated ECs. * p < 0.05, ** p < 0.01, *** p < 0.001, **** p < 0.0001.

The expressions of methyltransferase and demethylase were detected by Western Blot. METTL3 started to increase from 4 h and remained at the expression level for 40 h. FTO were also up-regulated after 24 h (Fig. 4A), while ALKBH5 did not change during this process (Supplementary Fig. 1D). Generally speaking, these m6A regulators are altered through dynamic processes. METTL3 and METTL14 are mainly located in the nucleus where they could interact with WTAAL to form a complex [28]. To confirm the localization changes of METTL3 and METTL14, cytoplasm and nucleus proteins were extracted and detected by western blot. Both nucleus METTL3 and METTL14 were increased after 24 h (Fig. 4B), which suggested that LPS recruited METTL3 and METTL14 to the nucleus for m6A modification. The activity of m6A regulators will also affect the m6A modification. We extracted nucleus protein and measured methylase and demethylase activity with m6A Demethylase Assay Kit. After LPS treatment, there was an increase in methylase activity (Fig. 4C), however, demethylase activity didn’t change in LPS-treated cells (Fig. 4D). These data suggested increased methyltransferase is the driver of increased m6A RNA methylation upon LPS challenge.

METTL3 is the binding subunit of S-adenosylmethionine (SAM) and the only protein possessing the m6A catalytic core function, so we suppose that METTL3 plays an important role in LPS-induced m6A RNA methylation. To confirm the role of METTL3, we knocked down the expression of METTL3 with METTL3 siRNA which was confirmed by western blot (Fig. 4E). Knockdown of METTL3 reduced the m6A methylation level in LPS-treated cells (Fig. 4F), which validates the vital role of METTL3 in LPS-induced RNA methylation. To explore the cellular role of m6A RNA modification in the response of ECs induced by endotoxin, protein expression of ICAM1 and endothelial function were also evaluated by western blot and ECIS. Knockdown of METTL3 reduced LPS-increased ICAM1 expression (Fig. 4G), consistent with its m6A modulation levels (Fig. 1L–M). LPS-induced endothelial hyper-permebility [29,30] was also attenuated by METTL3 knockdown (Fig. 4H), indicating that METTL3 and m6A play vital roles in EC barrier regulation in response to LPS challenge.

In parallel with siRNA studies, we used METTL3 inhibitor STM2457 (STM) to selectively inhibit METTL3’s enzymatic activity [31]. STM significantly suppressed the m6A methylation level in LPS-treated cells (Fig. 4I). STM treatment also reduced LPS-increased ICAM1 expression (Fig. 4J). LPS-induced endothelial barrier disruption was alleviated by STM (Fig. 4K). Together, these data suggest that METTL3 is the driving factor of LPS-induced global m6A RNA methylation and METTL3 remains a functional hub of endotlehlial inflammation upon LPS challenge.

The m6A reader proteins can detect and bind to the m6A methylated sites, regulating multiple biological processes including mRNA stability and gene expression [32]. Among nine m6A readers, only IGF2BP2 was found to be increased after LPS treatment in RNA sequencing data (Supplementary Fig. 2A). We utilized an IGF2BP2 inhibitor, CWI1–2, to selectively inhibit IGF2BP2’s activity and found that it could reduce the LPS-induced up-regulation of ICAM1 expression (Supplementary Fig. 2B). These data confirmed that ICAM1-upregulated by LPS challenge is depdent on its selective m6A methylation.

METTL3 inhibitor attenuates the inflammatory responses in CLP and LPS in Mice.

**Fig. 5.**
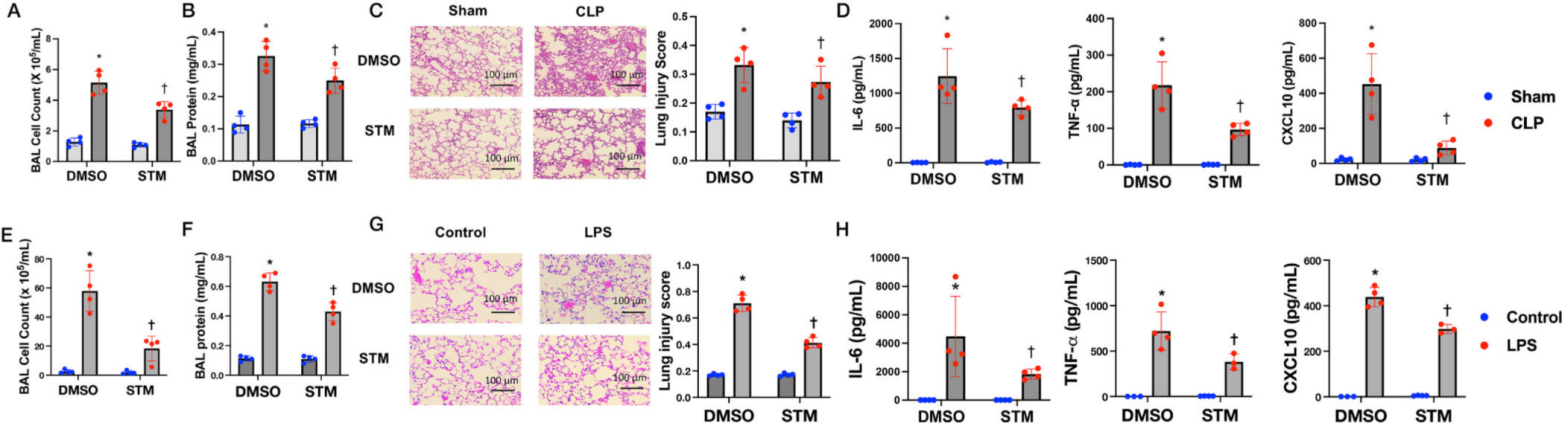
METTL3 inhibitor STM attenuates lung inflammation in mice receiving high tidal volume CLP and LPS. Wild-type C57BL/6 mice were intraperitoneally treated with 15 mg/kg STM before CLP or LPS challenge (24 h). Mice were harvested for lung inflammation analysis with (A) BAL cell count, (B) protein concentration, (C) lung injury score from histological analysis, and (D) BAL cytokines. * p < 0.05 compared to DMSO control, † p < 0.05 compared to DMSO with CLP. Wild-type C57BL/6 mice were intraperitoneally treated with 15 mg/kg STM two hours before LPS challenge (24 h). Mice were harvested for lung inflammation analysis with (E) BAL cell count, (F) protein concentration, (G) lung injury score from histological analysis, and (H) BAL cytokines. * p < 0.05 compared to DMSO control, † p < 0.05 compared to DMSO with CLP.

Next we examined the role of METTL3 in lung inflammation with METTL3 inhibitor STM in two preclinical models of acute lung injury. Acute lung inflammation in the CLP model was evaluated, and STM (15 mg/kg) significantly reduced the total BAL cell count and BAL protein in CLP mice (Fig. 5A–B). H&E staining showed infiltration of neutrophils in the CLP mice, and STM significantly attenuated the pulmonary pathological alterations in CLP mice (Fig. 5C). Moreover, the expression level of pro-inflammatory cytokines (TNFα, IL-1β, and CXCL10) were detected by ELISA. CLP substantially up-regulated the expression level of all these cytokines, while STM pretreatment decreased the expression level of these cytokines (Fig. 5D). In parallel, STM was also evaluated in a murine ALI model of pneumonia. C57BL/6 mice received STM (15 mg/kg) 2 h before LPS (1 mg/kg, i.t. instilation). STM significantly reduced LPS-induced BAL cell count and protein increases (Fig. 5E–F), the hallmark of experimental ALI. In addition, just like the effects in the CLP model, STM not only lessened the pulmonary inflammation in LPS-challenged mice (Fig. 5G), but also notably decreased LPS-increased cytokines levels of TNFα and CXCL10 (Fig. 5H). Our study demonstrates that STM2457 can suppress acute lung inflammation in both CLP and LPS models of ALI.

## 4. Discussion

This study provides a comprehensive characterization of the m6A RNA methylation landscape induced by LPS in lung endothelial cells, offering a deeper understanding of this crucial and emerging post-transcriptional modification during endotoxin challenge. Our findings confirm a significant increase in m6A methylation levels in lung endothelial cells upon LPS treatment. Specifically, our data reveal that: 1) m6A modification levels in CDS near the 5’ UTR are notably and selectively enhanced; 2) Increased m6A methylation correlates with elevated mRNA abundance; 3) m6A mRNA modification tends to favor pro-inflammatory genes, thereby promoting a sustained inflammatory response triggered by LPS; 4) Elevated expression of METTL3 emerges as a key factor driving the accumulation of m6A methylation induced by LPS; 5) METTL3 serves as a central pro-inflammatory regulator and holds potential as a novel therapeutic target for lung injury. These findings shed light on the intricate role of m6A RNA methylation in mediating inflammatory responses in lung endothelial cells and underscore the therapeutic potential of targeting METTL3 to mitigate lung injury.

Up to now, m6A RNA methylation is a typical and the only reversible modification that we know of. In this study, we reveal a critical role of METTL3-mediated m6A methylation in the endothelial barrier and trans-endothelial migration functions of ECs. The knockdown of METTL3 in ECs decreased ICAM1 protein expression and was accompanied by alleviating the destruction of endothelial barrier function caused by LPS. Moreover, inhibition of METTL3 also caused a reduction of ICAM1 and prevented LPS-induced endothelial barrier dysfunction. These findings from our study showed the potential role of METTL3 in regulating pulmonary endothelial cell m6A methylation and functionality.

This study focused on lung endothelial cells for the m6A landscape charaterization. Endothelial injury is indeed a critical initiating and propagating event in the pathogenesis of ALI. The pulmonary endothelium serves as a vital barrier between the vasculature and the alveolar space, and its integrity is essential for maintaining normal lung function. During ALI, injurious insults, including infection or trauma, can directly damage vascular endothelium, leading to endothelial dysfunction. This dysfunction manifests as increased vascular permeability, resulting in the leakage of protein-rich fluid into the alveoli, a hallmark of ALI. Furthermore, injured endothelial cells actively participate in the inflammatory cascade by releasing pro-inflammatory cytokines, chemokines, and adhesion molecules, thereby recruiting and activating immune cells and further exacerbating lung inflammation. Given this central role of endothelial injury in disrupting vascular barrier function and amplifying inflammation in ALI, we chose to focus our initial investigation on the mechanisms within these cells. Understanding the specific molecular events occurring within the lung endothelium is a crucial first step in developing targeted therapeutic strategies. Future studies will undoubtedly expand our investigation to encompass the roles of these other critical cell populations and their interactions with endothelial cells in the complex lung microenvironment comprised of dozens of cell types.

METTL3 is the catalytical monomer among all components in m6A methyltransferase complex. We found a positive correlation between m6A methylation level and mRNA expression and methylase activity of METTL3 in ECs. Further analysis revealed that METTL14, along with “m6A eraser” (FTO and ALKBH5) remained unalterable. Knockdown or inhibition of METTL3 protein expression resulted in reducing m6A methylation levels in LPS-treated ECs. These results revealed the possible functions of METTL3 in regulating pulmonary endothelial cell m6A methylation and functionality. As such, we examined a METTL3 inhibitor in ARDS mice to investigate the effects of METTL3-mediated m6A methylation on endothelial cells. Nevertheless, we did not rule out the regulatory roles of other m6A regulators for m6A modification in ECs, which still require further study. While effects of METTL3 inhibition on endothelial cells should also be considered when assessing METTL3 as a therapeutic target for ARDS therapy.

Our study suggests that AGE-RAGE signaling pathway was enriched among increased m6A modification genes according to KEGG analysis, while ICAM1 m6A methylation level was also increased. Both ICAM1 and AGE-RAGE pathways play an important role in the activated pulmonary endothelial cells, whereas they differentially and cooperatively regulate leukocyte adhesion and transmigration during acute inflammation [33,34]. The leukocyte adhesion and transmigration to vascular endothelium is a sign of the acute inflammatory process. Our study revealed that m6A methylation might regulate the leukocyte adhesion and transmigration directly.

The barrier function of molecules and cells across the pulmonary vessel wall, pulmonary endothelial cells play a critical role in mediating mediate key processes involved in lung homeostasis and modulating inflammation and cellular immunity. Many of these functions are damaged in ARDS. The MAPK signaling pathway is a major pathway that controls several biological roles of pulmonary endothelial cells in response to stimuli which includes growth factors, stress, and cytokines [35]. Our study revealed that m6A methylation levels are associated with MAPK signaling pathway protein expression level. Reducing m6A methylation level via METTL3 inhibitor could alleviate the inflammatory response in the LPS-induced mice shock model, indicating that m6A plays a critical role in reducing inflammation of endothelial cells.

In conclusion, our study confirms a METTL3-dependent m6A modification level is significantly increased in pulmonary endothelial cells after LPS treatment. METTL3-mediated mRNA methylation is enriched in pro-inflammatory genes to drive an inflammatory phenotype in LPS-challenged ECs. METTL3 is an emerging and effective therapeutic target for experimental ARDS.

## Supplementary Material

Supplementary Fig. 1. GSEA analysis of the NOD-like receptor (A) and apoptosis signaling pathway (B) on the gene expression matrix. C: m6A consensus sequence. D: Protein expression of m6A regulators (ALKBH5) in LPS-treated cells.

Supplementary Fig. 2. A. Gene expression of m6A readers in LPS-treated cells. B. ICAM1 expression in ICAM1 inhibitor (CWI1–2)-treated ECs.

Supplementary Tables

Supplementary data to this article can be found online at https://doi.org/10.1016/j.bbadis.2025.167907.

## Data Availability

Data will be made available on request.

## Abbreviations:

ALI: Acute Lung Injury
ECL: endothelial cell lining
FTO: obesity-associated protein
GSEA: Gene set enrichment analysis
ECs: endothelial cells
LPS: lipopolysaccharide
METTL3: methyltransferase like 3
METTL14: methyltransferase like 14
m6A: N6-methyladenosine
