# Supplementary figures and images for: Endotoxin-induced m6A RNA methylation landscape in lung endothelial cells: role of METTL3 in regulating inflammation and injury during acute lung injury
Source: Biochim Biophys Acta Mol Basis Dis. Author manuscript; Available in PMC 2025 Nov 13. (PMC12614311; doi:10.1016/j.bbadis.2025.167907)

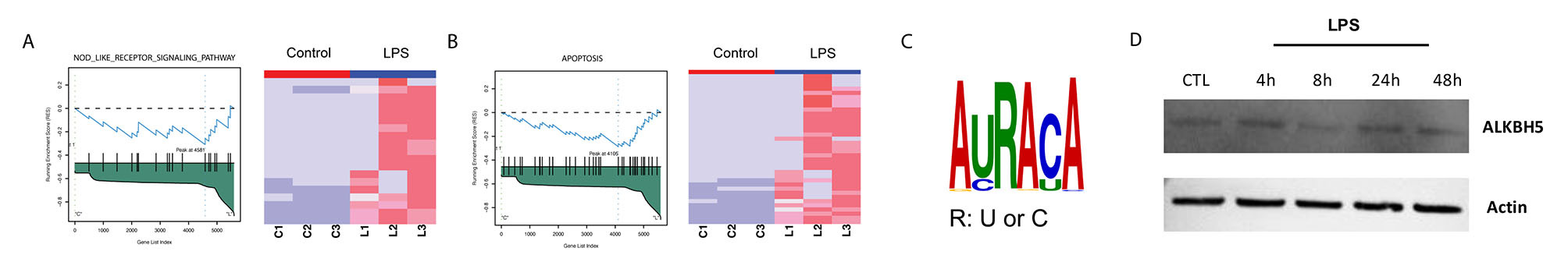

Supplement: Supplementary Fig. 1. GSEA analysis of the NOD-like receptor (A) and apoptosis signaling pathway (B) on the gene expression matrix. C: m6A consensus sequence. D: Protein expression of m6A regulators (ALKBH5) in LPS-treated cells. [file NIHMS2112530-supplement-Supplementary_Fig__1__GSEA_analysis_of_the_NOD-like_receptor__A__and_apoptosis_signaling_pathway__B__on_the_gene_expression_matrix__C__m6A_consensus_sequence__D__Protein_expression_of_m6A_regulators__ALKBH5__in_LPS-tre.jpg]

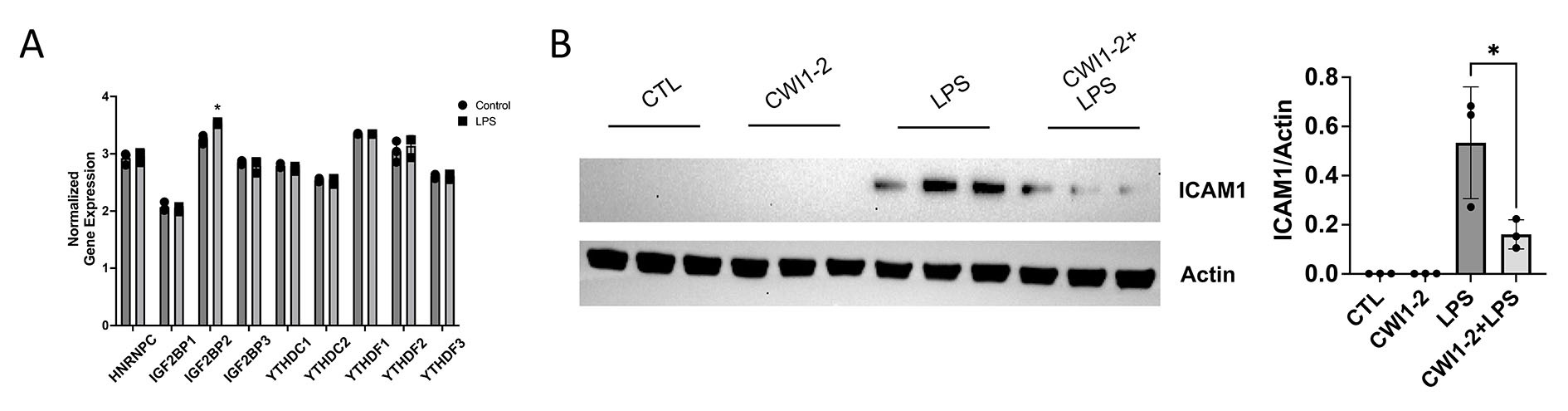

Supplement: Supplementary Fig. 2. A. Gene expression of m6A readers in LPS-treated cells. B. ICAM1 expression in ICAM1 inhibitor (CWI1–2)-treated ECs. [file NIHMS2112530-supplement-Supplementary_Fig__2__A__Gene_expression_of_m6A_readers_in_LPS-treated_cells__B__ICAM1_expression_in_ICAM1_inhibitor__CWI1_2_-treated_ECs_.jpg]
